# Appearance of NS3 Q80K mutation in HCV genotype 1a mono- or HIV/HCV co-infected patients in a Berlin laboratory

**DOI:** 10.7448/IAS.17.4.19741

**Published:** 2014-11-02

**Authors:** Robert Ehret, Stefan Neifer, Hauke Walter, Axel Baumgarten, Martin Obermeier

**Affiliations:** 1 Laboratory, MIB, Berlin, Germany; 2 Laboratory, Medical Laboratory Neifer, Berlin, Germany; 3 Out Patients Clinic, MIB, Berlin, Germany

## Abstract

**Introduction:**

Simeprevir, a new oral NS3/4A protease inhibitor, was recently approved by the FDA and the EMA for the treatment of patients with chronic HCV genotype 1, 4, 5 and 6 infection l. It has been recommended in the 2014 UK Consensus Guidelines as a possible treatment of previously untreated genotype 1a-infected patients. The antiviral efficacy of simeprevir is adversely affected by the mutation at the Q80K loci. There is controversial discussion that the incidence of Q80K in the European HCV 1a-infected community is very low and therefore testing of Q80K before starting a therapy including simeprevir is not necessary. We analyzed the appearance of Q80K in all sequenced HCV NS3A samples in 2014 in our laboratory.

**Materials and Methods:**

All in 2014 received orders for HCV resistance tests were analyzed with an in-house bulk sequencing method analyzing NS3A amino acids 1–181. Analysis was performed using geno2pheno HCV. The genotype 1a samples were selected, Q80K status and data of HIV co-infection were collected.

**Results:**

Forty-two HCV 1a samples were sent to us for resistance analyses from nine different medical centres in Berlin and Hannover, Germany. Nineteen (or 45%) of the sequences showed a Q80K mutation. Six extra clade I viruses had no Q80K mutation. Comparison between mono- and HIV-1 co-infected patients showed no difference in frequency of Q80K (mono-infected: 8 out of 19 patients; co-infected: 9 out of 23). For two 80K-positive patients, the HIV-status was not available.

**Conclusions:**

The incidence for Q80K mutation in HCV genotype 1a with overall 45% is substantially high in our cohort and does not differ between mono- and HIV-1 co-infected patients. Response to simeprevir is affected by the presence of viral Q80K. When treating HCV-infected patients with a simeprevir containing regimen, it is therefore important that HCV does not contain the Q80K mutation.

